# Effect of Magnesium Ion on the Radical-Scavenging Rate of Pterostilbene in an Aprotic Medium: Mechanistic Insight into the Antioxidative Reaction of Pterostilbene

**DOI:** 10.3390/antiox11020340

**Published:** 2022-02-09

**Authors:** Ikuo Nakanishi, Yoshimi Shoji, Kei Ohkubo, Megumi Ueno, Kei Shimoda, Ken-ichiro Matsumoto, Kiyoshi Fukuhara, Hiroki Hamada

**Affiliations:** 1 Quantum RedOx Chemistry Group, Institute for Quantum Life Science (iQLS), Quantum Life and Medical Science Directorate, National Institutes for Quantum Science and Technology (QST), Inage-ku, Chiba 263-8555, Japan; shoji.yoshimi@qst.go.jp; 2 Quantitative RedOx Sensing Group, Department of Radiation Regulatory Science Research, National Institute of Radiological Sciences (NIRS), Quantum Life and Medical Science Directorate, National Institutes for Quantum Science and Technology (QST), Inage-ku, Chiba 263-8555, Japan; ueno.megumi@qst.go.jp (M.U.); matsumoto.kenichiro@qst.go.jp (K.-i.M.); 3 Institute for Advanced Co-Creation Studies, Open and Transdisciplinary Research Initiatives, Osaka University, 2-8 Yamada-oka, Suita, Osaka 565-0871, Japan; 4 Department of Biomedical Chemistry, Faculty of Medicine, Oita University, Yufu City, Oita 879-5593, Japan; shimoda@oita-u.ac.jp; 5 School of Pharmacy, Showa University, Shinagawa-ku, Tokyo 142-8555, Japan; 6 Department of Life Science, Okayama University of Science, Kita-ku, Okayama 700-0005, Japan

**Keywords:** antioxidant, hydrogen transfer, resveratrol, pterostilbene, galvinoxyl, magnesium ion, kinetic isotope effect, density functional theory, cyclic voltammetry

## Abstract

Pterostilbene (PTS), a methylated analog of resveratrol (RSV), has recently attracted much attention due to its enhanced bioavailability compared to RSV. However, little is known about the radical-scavenging mechanism of PTS. In this study, we investigated the effect of Mg(ClO_4_)_2_ on the scavenging reaction of galvinoxyl radical (GO^•^) by PTS in acetonitrile (MeCN). GO^•^ was used as a model for reactive oxygen radicals. The second-order rate constant (*k*_H_) for the GO^•^-scavenging reaction by PTS was more than threefold larger than that by RSV, although thermodynamic parameters, such as the relative O–H bond dissociation energies of the phenolic OH groups, ionization potentials, and HOMO energies calculated by the density functional theory are about the same between PTS and RSV. The oxidation peak potential of PTS determined by the cyclic voltammetry in MeCN (0.10 M Bu_4_NClO_4_) was also virtually the same as that of RSV. On the other hand, no effect of Mg (ClO_4_)_2_ on the *k*_H_ values was observed for PTS, in contrast to the case for RSV. A kinetic isotope effect of 3.4 was observed when PTS was replaced by a deuterated PTS. These results suggest that a one-step hydrogen-atom transfer from PTS to GO^•^ may be the rate-determining step in MeCN.

## 1. Introduction

**Figure 1:**
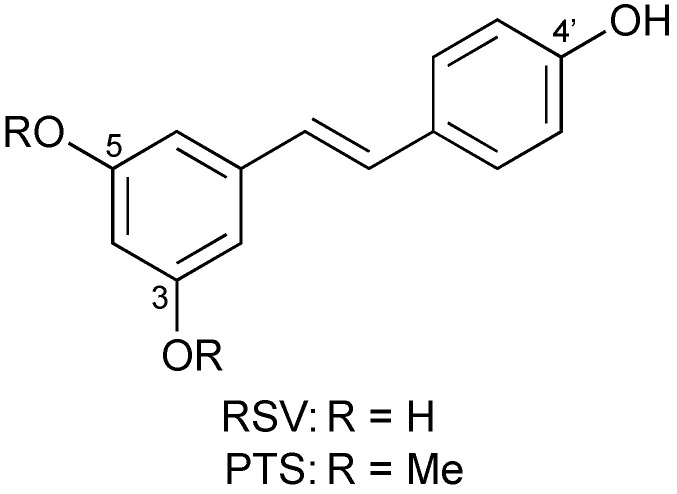
Chemical structures of RSV and PTS.

Resveratrol (RSV) (Figure 1), 3,5,4′-trihydroxy-*trans*-stilbene, found in grapes, is one of the representative antioxidants that shows a plethora of remarkable biological properties [1]. On the other hand, pterostilbene (PTS) (Figure 1), where the phenolic OH groups at the C-3 and C-5 positions in RSV are replaced by methoxy groups, has recently attracted much attention due to its enhanced bioavailability, compared to RSV [2,3,4,5,6]. However, little is known about the radical-scavenging mechanism of PTS. There are three mechanisms for the initial step of the radical-scavenging reaction of phenolic antioxidants: one-step hydrogen-atom transfer (HAT) from the phenolic OH group; electron transfer followed by proton transfer (ET–PT); and sequential proton-loss electron transfer (SPLET) [7]. Redox-inactive metal ions, such as magnesium ion (Mg^2+^), are a powerful tool to examine the involvement of electron-transfer processes [8,9,10,11,12,13], because it has been reported that electron-transfer reactions are significantly accelerated by their presence [14,15]. The stabilization of one-electron reduced species by these metal ions leads to the acceleration. In fact, a significant acceleration in the radical-scavenging reaction of RSV or (+)-catechin was observed in the presence of Mg^2+^ in acetonitrile (MeCN), suggesting that the reaction may proceed via the ET–PT mechanism [8,12]. In MeCN, an aprotic solvent, the SPLET mechanism can be ruled out, since the deprotonation of the phenolic OH group hardly occurs.

On the other hand, no effect of Mg^2+^ was observed on the radical-scavenging rate of a vitamin E analog in MeCN, suggesting that the reaction may proceed via the HAT mechanism [13]. The elucidation of the detailed radical-scavenging mechanism is of considerable importance for the development of synthetic antioxidants having a stronger activity than naturally occurring ones. In fact, we have previously reported a number of synthetic antioxidants that show a stronger radical-scavenging activity than the parent compounds [16,17,18,19,20,21,22,23,24,25,26,27,28,29,30,31]. We report herein the effect of Mg^2+^ on the scavenging reaction of galvinoxyl radical (GO^•^) by PTS in MeCN. GO^•^ is frequently used as a model for the reactivity of reactive oxygen species to evaluate the activity of antioxidants [32]. The difference in the effect of Mg^2+^ on the GO^•^-scavenging rates between RSV and PTS provides a valuable insight into the structure–activity and structure–mechanism relationships for the radical-scavenging reaction of phenolic antioxidants.

## 2. Materials and Methods

### 2.1. Materials

PTS (analytical grade) was commercially obtained from Excel Chemical Co., Ltd., Tokyo, Japan, and used without further purification. GO^•^ was purchased from the Tokyo Chemical Industry Co., Ltd., Tokyo, Japan. MeCN (spectral grade) used as a solvent was commercially obtained from Nacalai Tesque, Inc., Kyoto, Japan, and used as received. Mg (ClO_4_)_2_ was purchased from FUJIFILM Wako Pure Chemical Corporation, Osaka, Japan. Deuterated PTS was prepared by dissolving 1 g of PTS in 20 mL of 10% D_2_O in CD_3_OD and then removing the solvent by evaporation, a process that was repeated three times (99% deuteration degree for NMR spectroscopy). RSV was commercially obtained from Sigma-Ardrich, St. Louis, MO, USA). Tetra-*n*-butylammonium perchlorate (Bu_4_NClO_4_), used as a supporting electrolyte for electrochemical measurements, was purchased from the Tokyo Chemical Industry Co., Ltd., Japan, recrystallized from ethanol (spectral grade, Nacalai Tesque, Inc., Kyoto, Japan), and dried under vacuum at 313 K.

### 2.2. Spectral and Kinetic Measurements

UV-vis spectra were recorded on an Agilent 8453 photodiode array spectrophotometer (Agilent Technologies, Santa Clara, CA, USA). The GO^•^-scavenging rates of PTS in MeCN were determined based on the absorbance change at 428 nm due to GO^•^ (*ε* = 1.4 × 10^5^ M^−1^ cm^−1^) every 1.5 s after mixing of 0.2 mL of an MeCN solution of GO^•^ (2.0 × 10^−5^ M) with 0.2 mL of an MeCN solution containing PTS (3.0 × 10^−3^, 5.9 × 10^−3^, 8.9 × 10^−3^, and 1.2 × 10^−2^ M), using a stopped-flow technique on a UNISOKU RSP-1000-02NM spectrophotometer (UNISOKU Co., Ltd., Osaka, Japan), which was thermostated at 298 K with a Thermo Scientific NESLAB RTE-7 Circulating Bath (Thermo Fisher Scientific, Inc., Waltham, MA, USA). Pseudo-first-order rate constants (*k*_obs_) were determined by a least-square curve fit on an Apple MacBook Pro personal computer (Apple Inc., Cupertino, CA, USA). The first-order plots of ln (*A*–*A*_∞_) vs. time (*A* and *A*_∞_ are the absorbance at the reaction time and the final absorbance, respectively) were linear until three or more half-lives with the correlation coefficient *ρ* > 0.999. It was confirmed that the *k*_obs_ values derived from at least three independent measurements agreed within an experimental error of ±5% in each case.

### 2.3. Theoretical Calculations

Density functional theory (DFT) calculations were performed on a 16-processor high performance computer (ForScientist XD1, HPC System Inc., Tokyo, Japan). The geometry optimization was performed using the M06-2X/6-31++G(d) basis set with a C-PCM solvation model parameterized for acetonitrile, as implemented in the Gaussian 09 (Revision A.02, Gaussian, Inc., Wallingford, CT, USA) (the computational details (coordinates and energy values) are available in the Supplementary Materials) [33]. The calculated energy difference values (*D*_HT_, HT: hydrogen transfer) are defined as the energy difference values of heat of formations of phenoxyl radicals (Δ*H* (phenoxyl radical)) and the parent phenols (Δ*H* (phenol)) (Equation (1)), while the bond dissociation energy (BDE) includes the heat of formation of hydrogen atom (Δ*H* (H^•^)) (Equation 2). The ionization potentials (IP) were calculated from the energy difference between the phenol and the corresponding radical cation.

*D*_HT_ = Δ*H*(phenoxyl radical) − Δ*H*(phenol) (1)

BDE = *D*_HT_ + Δ*H*(H^•^) (2)

### 2.4. Electrochemical Measurements

The cyclic voltammetry measurements were performed on an ALS-630A electrochemical analyzer (BAS Co. Ltd., Tokyo, Japan) in MeCN containing 0.10 M Bu_4_NClO_4_ as a supporting electrolyte. The Pt working electrode (BAS Co. Ltd., Tokyo, Japan) was polished with an alumina polishing suspension (BAS Co. Ltd., Tokyo, Japan) and an alumina polishing pad (BAS Co. Ltd., Tokyo, Japan) and rinsed with methanol (FUJIFILM Wako Pure Chemical Corporation, Osaka, Japan) before use. The counter electrode was a platinum wire (BAS Co. Ltd., Tokyo, Japan). The concentration of PTS or RSV was 1.0 × 10^−3^ M. The measured potentials were recorded with respect to an Ag/AgNO_3_ (0.01 M) reference electrode (BAS Co. Ltd., Tokyo, Japan) with the sweep rate of 100 mV s^−1^ at 298 K. The oxidation peak potentials (*E*_pa_) were converted to those vs. the saturated calomel electrode (SCE) by adding 0.29 V [34].

## 3. Results and Discussion

**Figure 2:**
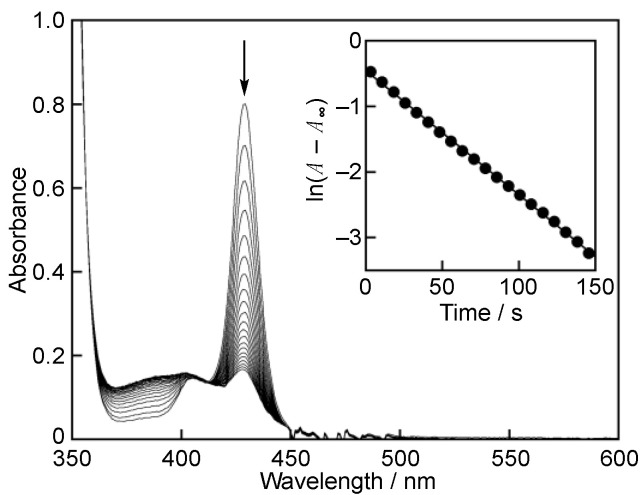
Spectral change observed every 7.5 s (once every fifth measurement) during the reaction of PTS (1.5 × 10^−3^ M) with GO^•^ (1.0 × 10^−5^ M) in MeCN at 298 K. Inset: the first-order plot of the absorbance at 428 nm.

**Figure 3:**
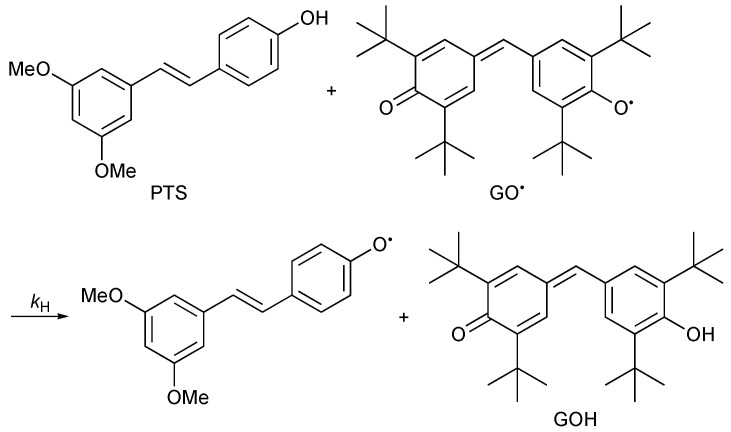
Hydrogen transfer from PTS to GO^•^.

**Figure 4:**
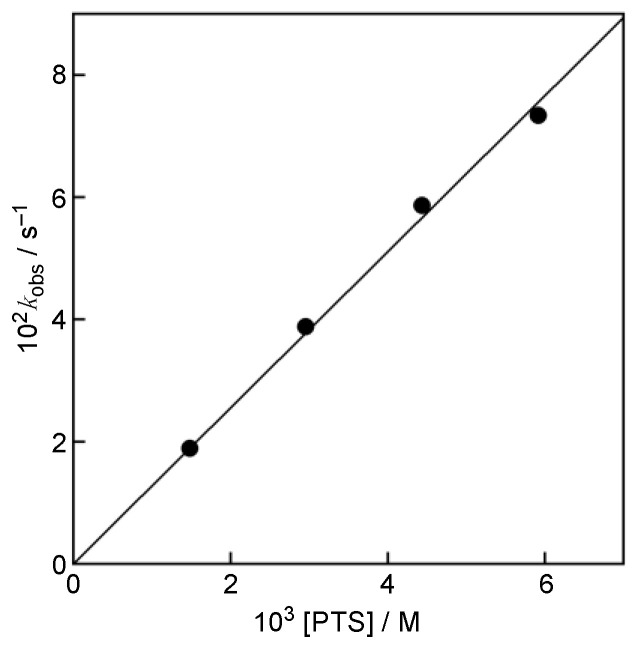
Plots of *k*_obs_ vs. concentrations of PTS.

The rate of the reaction of PTS with GO^•^ was investigated by monitoring the spectral change during the reaction. Upon mixing of PTS with GO^•^ in MeCN, the absorption band at 428 nm due to GO^•^ decreased gradually, as shown in Figure 2. This indicates that PTS efficiently scavenged GO^•^ (Figure 3). The decay of the absorbance at 428 nm monitored by a stopped-flow technique obeyed pseudo-first-order kinetics, when the concentration of PTS ([PTS]) was maintained at more than a 10-fold excess of the concentration of GO^•^ (inset of Figure 2). The pseudo-first-order rate constants (*k*_obs_) linearly increased with increasing [PTS] (Figure 4). The second-order rate constant (*k*_H_) in Equation 3 could be determined from the slope of the plot (Equation 4) for the GO^•^-scavenging reaction by PTS (Figure 3) in MeCN to be 1.3 × 10 M^−1^ s^−1^. This *k*_H_ value is more than threefold larger than that obtained for RSV (*k*_H_ = 4.1 M^−1^ s^−1^) under the same experimental conditions (Table 1) [8].

−d[GO^•^]/d*t* = *k*_H_[PTS][GO^•^] (3)

*k*_obs_ ([PTS] > 10[GO^•^]) = *k*_H_[PTS] (4)

**Figure 5:**
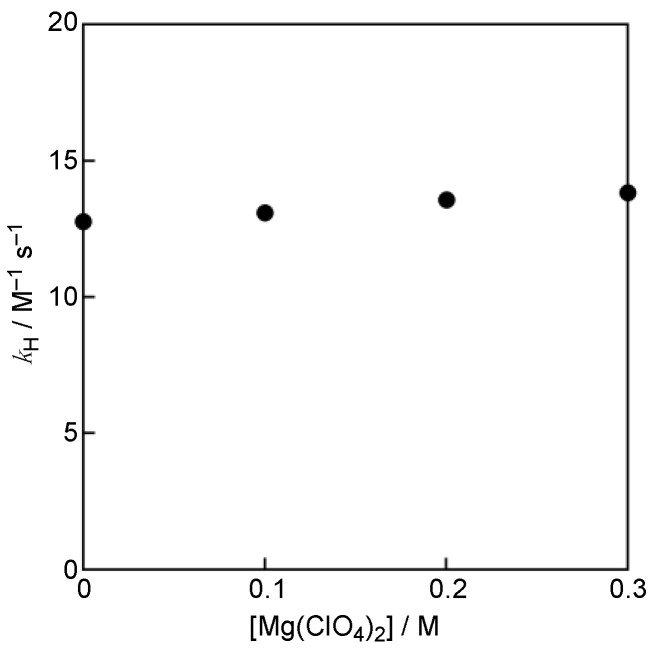
Plots of *k*_H_ vs. concentrations of Mg(ClO_4_)_2_.

The *k*_H_ values were also determined for the reaction of PTS with GO^•^ in the presence of Mg(ClO_4_)_2_ to examine whether the electron-transfer reaction is involved as the rate-determining step. In the case of RSV, the *k*_H_ values were reported to increase with increasing the concentration of Mg(ClO_4_)_2_ [8]. Such an acceleration by Mg(ClO_4_)_2_ indicates that the GO^•^-scavenging reaction by RSV proceeds via an electron transfer from RSV to GO^•^ as the rate-determining step in MeCN [8]. On the other hand, no acceleration effect of Mg(ClO_4_)_2_ was observed on the *k*_H_ values for PTS, as shown in Figure 5. Thus, the GO^•^-scavenging reaction by PTS may proceed via the one-step hydrogen-atom transfer rather than via the electron transfer followed by proton transfer as the rate-determining step in MeCN.

The deuterium primary kinetic isotope effect (KIE) was investigated with the use of a deuterated pterostilbene (PTS-*d*), which was prepared by the exchange reaction of H^+^ with D^+^ in the phenolic OH group of PTS in deuterated methanol (CH_3_OD). The second-order rate constant (*k*_D_) for the reaction of PTS-*d* with GO^•^ was determined to be 3.7 M^−1^ s^−1^. Thus, the KIE value (*k*_H_/*k*_D_) was calculated to be 3.4. A KIE value larger than unity (*k*_H_/*k*_D_ > 1) clearly supports the involvement of a hydrogen-atom transfer as the rate-determining step [35].

The density functional theory (DFT) calculations were also carried out to compare the radical-scavenging reactivity of PTS with RSV [33]. The energy difference values (*D*_HT_, HT: hydrogen transfer) between the parent phenols (RSV and PTS) and the corresponding phenoxyl radicals were determined by DFT calculation at the M06-2X/6-31++G(d) level with C-PCM = acetonitrile. The *D*_HT_ values reflect the relative O–H bond dissociation energies of the phenolic OH groups. Thus, the hydrogen-atom donor ability of the phenolic OH groups can be evaluated by the *D*_HT_ values. The calculated *D*_HT_ value thus obtained for the OH group at the C-4′ position in PTS (398.2 kcal mol^−1^) is about the same as that in RSV (398.4 kcal mol^−1^) (Table 1). On the other hand, the electron donor ability of the phenols can be evaluated by their ionization potentials (IP). The IP value of PTS was also calculated by DFT calculation to be 132.6 kcal mol^−1^, which is also about the same as that of RSV (133.3 kcal mol^−1^) (Table 1). The HOMO energies (*E*_HOMO_) of PTS and RSV were also calculated by the DFT calculations, as shown in Table 1, and were also almost the same. The energy difference of the HOMO energies (0.8 kcal mol^−1^) is virtually the same as that of the IP values (0.8 kcal mol^−1^).

**Figure 6:**
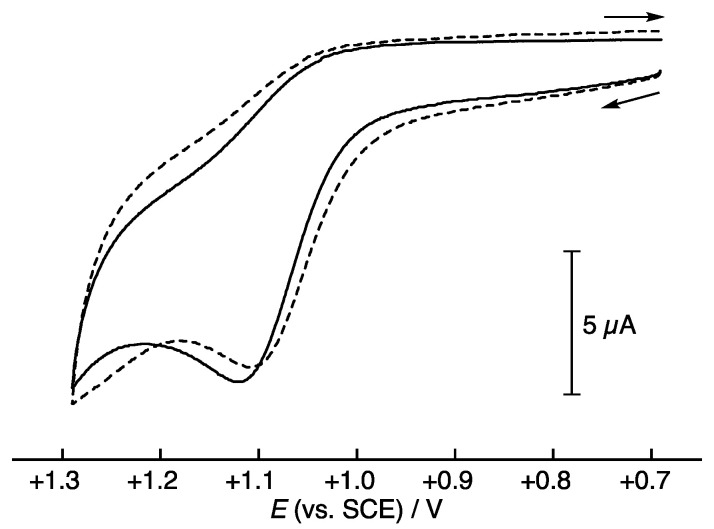
Cyclic voltammograms of PTS (1.0 × 10^−3^ M) (solid line) and RSV (1.0 × 10^−3^ M) (dashed line) in MeCN (0.10 M Bu_4_NClO_4_) recorded at the scan rate of 100 mV s^−1^ on a Pt working electrode.

The electron donor ability of PTS was also compared with RSV by cyclic voltammetry measurements in MeCN containing 0.10 M Bu_4_NClO_4_ as a supporting electrolyte. An irreversible oxidation (anordic) peak was observed both for PTS and RSV, as shown in Figure 6. From the cyclic voltammograms, the oxidation peak potentials (*E*_pa_) of PTS and RSV were determined to be +1.11 and +1.10 V vs. SCE at the scan rate of 100 mV s^−1^ (Table 1). These *E*_pa_ values are also virtually the same, indicating that the electron donor ability of PTS is almost the same as that of RSV in MeCN (0.10 M Bu_4_NClO_4_).

The partition coefficient of PTS (log *P* = 2.69) is significantly larger than that of RSV (log *P* = 2.048) [3]. Thus, the hydrophobic interaction between PTS and GO^•^ is thought to be stronger than that between RSV and GO^•^ in MeCN, an aprotic polar solvent, although such an interaction could not be observed experimentally. This may cause the faster GO^•^-scavenging rate for PTS compared to RSV.

## 4. Conclusions

PTS scavenged GO^•^ more rapidly than RSV. The methyl substitution of the phenolic OH groups in RSV may result in the change in the GO^•^-scavenging mechanism from electron transfer to the one-step hydrogen-atom transfer, providing valuable information about the structure–reactivity and structure–mechanism relationships for the radical-scavenging reaction of phenolic antioxidants.

## Figures and Tables

**Table 1:** Experimental *k*_H_ values in MeCN, calculated *D*_HT_, IP and *E*_HOMO_ values by DFT, and experimental *E*_pa_ values in MeCN (0.10 M Bu_4_NClO_4_) for RSV and PTS.

Compound	*k*_H_/M^−1^ s^−1^	*D*_HT_/ kcal mol^−1^	IP/kcal mol^−1^	*E*_HOMO_/kcal mol^−1^	*E*_pa_/V vs. SCE
RSV	4.1	398.4	133.3	–157.7	+1.10
PTS	1.3 × 10	398.2	132.6	–158.9	+1.11

## Data Availability

Data is contained within the article.

## Supplementary Materials

The following are available online at https://www.mdpi.com/article/10.3390/antiox11020340/s1. The computational details (coordinates and energy values).
