# Supplementary material for: Effect of Magnesium Ion on the Radical-Scavenging Rate of Pterostilbene in an Aprotic Medium: Mechanistic Insight into the Antioxidative Reaction of Pterostilbene
Source: Antioxidants (Basel). 2022 Feb 9;11(2):340. doi: 10.3390/antiox11020340 (PMC8868536; doi:10.3390/antiox11020340)
Supplement: Supplementary file 1 [file antioxidants-11-00340-s001.zip › antioxidants-1574936-supplementary.pdf]

Coordinates

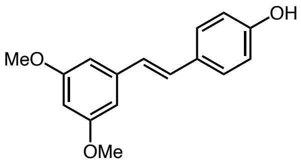

M06-2X/6-31++G(d) level with C-PCM = acetonitrile
Charge = 0
Spin: Singlet

Center Number	Atomic Number	Atomic Type	Coordinates (Angstroms) X	Y	Z
1	6	0	–1.151004	0.433177	–0.091654
2	6	0	–0.177496	–0.472734	0.101789
3	6	0	1.270488	–0.204634	0.062899
4	6	0	2.136699	–1.302796	–0.024218
5	6	0	3.518769	–1.092416	–0.078723
6	6	0	4.038741	0.199368	–0.042445
7	6	0	3.170342	1.288096	0.056889
8	6	0	1.788259	1.098212	0.113766
9	1	0	1.118032	1.942305	0.216310
10	1	0	1.719270	–2.302720	–0.055823
11	6	0	–2.597780	0.178848	–0.041318
12	6	0	–3.153927	–1.010796	0.454335
13	6	0	–4.528651	–1.213545	0.470737
14	6	0	–5.384963	–0.216100	–0.005336
15	6	0	–4.857505	0.980081	–0.492848
16	6	0	–3.480486	1.167628	–0.504102
17	1	0	–3.077437	2.102846	–0.885607
18	1	0	–2.510349	–1.793059	0.846289
19	8	0	–6.740753	–0.353868	–0.009687
20	1	0	–6.992854	–1.213839	0.361121
21	8	0	3.769051	2.509071	0.101523
22	8	0	4.434844	–2.094090	–0.165667
23	1	0	–4.938947	–2.141309	0.862026
24	1	0	–5.532418	1.749306	–0.855602
25	1	0	5.111466	0.358923	–0.077990
26	1	0	–0.880463	1.461152	–0.331973
27	1	0	–0.442841	–1.513792	0.281868
28	6	0	3.958082	–3.430678	–0.194094
29	1	0	3.401236	–3.668401	0.718687
30	1	0	3.322648	–3.602722	–1.069636
31	1	0	4.843673	–4.062048	–0.255776
32	6	0	2.938074	3.652782	0.225400
33	1	0	2.258682	3.738967	–0.629630
34	1	0	2.359174	3.618708	1.154721
35	1	0	3.610121	4.509837	0.245208

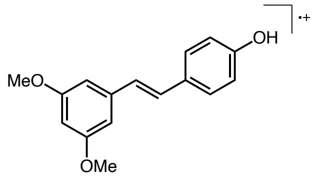

M06-2X/6-31++G(d) level with C-PCM = acetonitrile
Charge = 1
Spin: Doublet

Center Number	Atomic Number	Atomic Type	Coordinates (Angstroms) X	Y	Z
1	6	0	1.176929	0.453642	–0.000399
2	6	0	0.164780	–0.490548	–0.000112
3	6	0	–1.241166	–0.207251	–0.000060
4	6	0	–2.117713	–1.315001	–0.000103
5	6	0	–3.493345	–1.098140	–0.000058
6	6	0	–3.992083	0.208422	0.000059
7	6	0	–3.122092	1.307792	0.000143
8	6	0	–1.746090	1.113336	0.000079
9	1	0	–1.071853	1.958541	0.000179
10	1	0	–1.701857	–2.315555	–0.000189
11	6	0	2.565009	0.175239	–0.000253
12	6	0	3.110575	–1.145133	0.000470
13	6	0	4.465454	–1.347231	0.000646
14	6	0	5.337390	–0.231316	0.000115
15	6	0	4.828356	1.087422	–0.000616
16	6	0	3.472391	1.278265	–0.000785
17	1	0	3.069053	2.286600	–0.001341
18	1	0	2.455470	–2.008810	0.000897
19	8	0	6.657184	–0.361841	0.000274
20	1	0	6.931729	–1.295549	0.000798
21	8	0	–3.722900	2.517980	0.000298
22	8	0	–4.423292	–2.077636	–0.000112
23	1	0	4.877333	–2.351857	0.001212
24	1	0	5.525438	1.918186	–0.001025
25	1	0	–5.064738	0.374705	0.000108
26	1	0	0.909608	1.507310	–0.000780
27	1	0	0.429299	–1.544756	0.000066
28	6	0	–3.968700	–3.425673	–0.000231
29	1	0	–3.377037	–3.635960	–0.897177
30	1	0	–3.376987	–3.636104	0.896647
31	1	0	–4.866621	–4.041077	–0.000255
32	6	0	–2.893428	3.674004	0.000413
33	1	0	–2.266282	3.701847	0.897506
34	1	0	–2.266311	3.702046	–0.896694
35	1	0	–3.571774	4.525425	0.000518

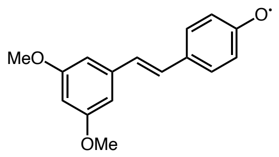

M06-2X/6-31++G(d) level with C-PCM = acetonitrile
Charge = 0
Spin: Doublet

Center Number	Atomic Number	Atomic Type	Coordinates (Angstroms) X	Y	Z
1	6	0	1.227862	0.442953	0.000156
2	6	0	0.228429	–0.482777	–0.000027
3	6	0	–1.205063	–0.201605	–0.000036
4	6	0	–2.077921	–1.301113	0.000043
5	6	0	–3.459316	–1.086089	0.000036
6	6	0	–3.968418	0.211106	–0.000055
7	6	0	–3.094885	1.301918	–0.000142
8	6	0	–1.713890	1.108384	–0.000141
9	1	0	–1.041157	1.955497	–0.000256
10	1	0	–1.664731	–2.303143	0.000134
11	6	0	2.634064	0.162232	0.000093
12	6	0	3.177985	–1.162462	–0.000212
13	6	0	4.524136	–1.372837	–0.000285
14	6	0	5.468191	–0.261341	–0.000069
15	6	0	4.897239	1.077498	0.000239
16	6	0	3.545458	1.263136	0.000316
17	1	0	3.135205	2.270136	0.000550
18	1	0	2.509605	–2.017558	–0.000386
19	8	0	6.701935	–0.451326	–0.000142
20	8	0	–3.689268	2.523075	–0.000264
21	8	0	–4.382720	–2.081531	0.000157
22	1	0	4.938809	–2.376685	–0.000516
23	1	0	5.586215	1.916924	0.000404
24	1	0	–5.041010	0.375451	–0.000054
25	1	0	0.969630	1.499897	0.000368
26	1	0	0.483990	–1.540092	–0.000173
27	6	0	–3.914772	–3.422671	–0.000025
28	1	0	–3.320074	–3.629775	–0.896052
29	1	0	–3.320065	–3.630025	0.895938
30	1	0	–4.805678	–4.048992	–0.000101
31	6	0	–2.852490	3.671046	0.000264
32	1	0	–2.223457	3.696363	0.896406
33	1	0	–2.223224	3.697030	–0.895696
34	1	0	–3.523285	4.528958	0.000480

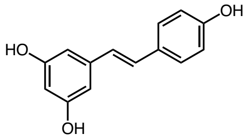

M06-2X/6-31++G(d) level with C-PCM = acetonitrile
Charge = 0
Spin: Singlet

Center Number	Atomic Number	Atomic Type	Coordinates (Angstroms) X	Y	Z
1	6	0	0.693910	0.460764	0.033887
2	6	0	–0.270347	–0.472985	–0.029793
3	6	0	–1.720708	–0.219971	–0.021335
4	6	0	–2.579176	–1.322333	0.076920
5	6	0	–3.962226	–1.132654	0.093004
6	6	0	–4.510851	0.143236	0.011848
7	6	0	–3.648983	1.235363	–0.090600
8	6	0	–2.266184	1.069423	–0.110919
9	1	0	–1.627053	1.942516	–0.206854
10	1	0	–2.168008	–2.326968	0.142736
11	6	0	2.143693	0.222967	0.011456
12	6	0	2.719200	–1.030990	–0.247742
13	6	0	4.097618	–1.206238	–0.248477
14	6	0	4.938125	–0.117891	0.004854
15	6	0	4.391231	1.140752	0.255699
16	6	0	3.010745	1.299795	0.255296
17	1	0	2.591425	2.284149	0.450059
18	1	0	2.089460	–1.889536	–0.462221
19	8	0	6.296188	–0.226669	0.013498
20	1	0	6.562689	–1.140496	–0.172656
21	8	0	–4.231643	2.465424	–0.175942
22	8	0	–4.836036	–2.174989	0.188494
23	1	0	4.523834	–2.185729	–0.452113
24	1	0	5.053704	1.979388	0.447356
25	1	0	–5.586062	0.287129	0.022730
26	1	0	0.412632	1.509699	0.123432
27	1	0	0.004649	–1.525660	–0.079781
28	1	0	–3.549940	3.153214	–0.228632
29	1	0	–4.351674	–3.013270	0.242965

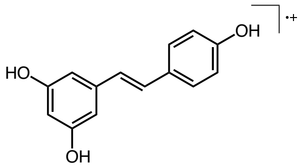

M06-2X/6-31++G(d) level with C-PCM = acetonitrile
Charge = 1
Spin: Doublet

Center Number	Atomic Number	Atomic Type	Coordinates (Angstroms) X	Y	Z
1	6	0	–0.718742	0.486819	0.000006
2	6	0	0.274057	–0.476667	–0.000101
3	6	0	1.687212	–0.219535	–0.000082
4	6	0	2.545991	–1.338386	–0.000041
5	6	0	3.923524	–1.147632	0.000030
6	6	0	4.456142	0.142705	0.000061
7	6	0	3.601513	1.251024	0.000008
8	6	0	2.223462	1.086527	–0.000074
9	1	0	1.581093	1.960377	–0.000145
10	1	0	2.129002	–2.341694	–0.000052
11	6	0	–2.110828	0.232137	0.000013
12	6	0	–2.677818	–1.080391	–0.000054
13	6	0	–4.034846	–1.260453	–0.000062
14	6	0	–4.889043	–0.129649	–0.000002
15	6	0	–4.358762	1.181835	0.000077
16	6	0	–3.000612	1.350556	0.000089
17	1	0	–2.580674	2.352060	0.000150
18	1	0	–2.036557	–1.954278	–0.000096
19	8	0	–6.209051	–0.239480	–0.000006
20	1	0	–6.499058	–1.168798	–0.000055
21	8	0	4.197774	2.468617	0.000019
22	8	0	4.812280	–2.170767	0.000082
23	1	0	–4.463730	–2.257854	–0.000113
24	1	0	–5.042552	2.023464	0.000128
25	1	0	5.531611	0.287975	0.000127
26	1	0	–0.430986	1.535053	0.000094
27	1	0	–0.009668	–1.525736	–0.000188
28	1	0	3.532444	3.174876	0.000148
29	1	0	4.353553	–3.025756	0.000025

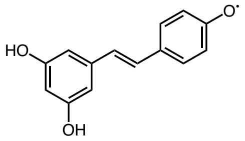

M06-2X/6-31++G(d) level with C-PCM = acetonitrile
Charge = 0
Spin: Doublet

Center Number	Atomic Number	Atomic Type	Coordinates (Angstroms) X	Y	Z
1	6	0	0.770367	0.472961	0.000052
2	6	0	–0.209674	–0.471572	–0.000015
3	6	0	–1.648060	–0.215307	–0.000013
4	6	0	–2.506709	–1.324619	0.000030
5	6	0	–3.888748	–1.133793	0.000042
6	6	0	–4.430681	0.148243	0.000011
7	6	0	–3.569723	1.246929	–0.000038
8	6	0	–2.187764	1.081391	–0.000053
9	1	0	–1.547291	1.957799	–0.000104
10	1	0	–2.095397	–2.330997	0.000056
11	6	0	2.182350	0.218180	0.000030
12	6	0	2.748664	–1.096839	–0.000074
13	6	0	4.098276	–1.283631	–0.000104
14	6	0	5.022436	–0.155532	–0.000049
15	6	0	4.428256	1.173306	0.000087
16	6	0	3.073330	1.335222	0.000117
17	1	0	2.645538	2.334891	0.000209
18	1	0	2.095049	–1.963277	–0.000130
19	8	0	6.259238	–0.323796	–0.000038
20	8	0	–4.154311	2.476579	–0.000080
21	8	0	–4.768221	–2.172562	0.000084
22	1	0	4.530609	–2.280041	–0.000181
23	1	0	5.102508	2.024552	0.000157
24	1	0	–5.505875	0.293116	0.000022
25	1	0	0.491551	1.524745	0.000127
26	1	0	0.064403	–1.524224	–0.000070
27	1	0	–3.476014	3.169846	–0.000073
28	1	0	–4.292652	–3.017821	0.000133

Total Energies (in hartree) of RSV and PTS

Compound	Neutral	Radical Cation	Phenoxyl Radical
RSV	–766.0912804	–765.8787068	–765.4564117
PTS	–844.6537267	–844.4425055	–844.0191963
